# Co-targeting the MAPK and PI3K/AKT/mTOR pathways in two genetically engineered mouse models of schwann cell tumors reduces tumor grade and multiplicity

**DOI:** 10.18632/oncotarget.1609

**Published:** 2014-01-20

**Authors:** Adrienne L. Watson, Leah K. Anderson, Andrew D. Greeley, Vincent W. Keng, Eric P. Rahrmann, Amanda L. Halfond, Natasha M. Powell, Margaret H. Collins, Tilat Rizvi, Christopher L. Moertel, Nancy Ratner, David A. Largaespada

**Affiliations:** ^1^ Masonic Cancer Center, University of Minnesota, Minneapolis, MN, USA; ^2^ Department of Genetics, Cell Biology and Development, University of Minnesota, Minneapolis, MN, USA; ^3^ Center for Genome Engineering, University of Minnesota, Minneapolis, MN, USA; ^4^ Brain Tumor Program, University of Minnesota, Minneapolis, MN, USA; ^5^ Health and Natural Sciences Department, University of Minnesota, Minneapolis, MN, USA; ^6^ Department of Pediatrics, University of Minnesota, Minneapolis, MN, USA; ^7^ Division of Experimental Hematology and Cancer Biology, Cincinnati Children's Hospital Medical Center, Cincinnati, OH, USA.; ^8^ Division of Pathology and Laboratory Medicine, Cincinnati Children's Hospital Medical Center, Cincinnati, OH, USA.; ^9^ Department of Pediatrics, Cincinnati Children's Hospital Medical Center, Cincinnati, OH, USA.; ^10^ Department of Applied Biology and Chemical Technology, The Hong Kong Polytechnic University, Hung Hom, Kowloon, Hong Kong.

**Keywords:** Malignant peripheral nerve sheath tumors, plexiform neurofibromas, Schwann cells, neurofibromatosis type 1 syndrome, neurofibromin 1, PI3K/AKT/mTOR signaling, MAPK signaling, targeted therapies, genetically engineered mouse models, combination therapy

## Abstract

Malignant peripheral nerve sheath tumors (MPNSTs) are soft tissue sarcomas that occur spontaneously, or from benign plexiform neurofibromas, in the context of the genetic disorder Neurofibromatosis Type 1 (NF1). The current standard treatment includes surgical resection, high-dose chemotherapy, and/or radiation. To date, most targeted therapies have failed to demonstrate effectiveness against plexiform neurofibromas and MPNSTs. Recently, several studies suggested that the mTOR and MAPK pathways are involved in the formation and progression of MPNSTs. Everolimus (RAD001) inhibits the mTOR and is currently FDA approved for several types of solid tumors. PD-0325901 (PD-901) inhibits MEK, a component of the MAPK pathway, and is currently in clinical trials. Here, we show in vitro than MPNST cell lines are more sensitive to inhibition of cellular growth by Everolimus and PD-901 than immortalized human Schwann cells. In combination, these drugs synergistically inhibit cell growth and induce apoptosis. In two genetically engineered mouse models of MPNST formation, modeling both sporadic and NF1-associated MPNSTs, Everolimus, or PD-901 treatment alone each transiently reduced tumor burden and size, and extended lifespan. However, prolonged treatment of each single agent resulted in the development of resistance and reactivation of target pathways. Combination therapy using Everolimus and PD-901 had synergistic effects on reducing tumor burden and size, and increased lifespan. Combination therapy allowed persistent and prolonged reduction in signaling through both pathways. These data suggest that co-targeting mTOR and MEK may be effective in patients with sporadic or NF1-associated MPNSTs.

## INTRODUCTION

Malignant peripheral nerve sheath tumors (MPNSTs) are soft tissue sarcomas that occur in 10% of patients with Neurofibromatosis Type 1 (NF1), but which can also occur spontaneously in the general population [1]. MPNSTs are derived from Schwann cells and/or their precursors and are highly associated with peripheral nerves [1]. The current standard of care for patients with MPNSTs includes surgery, radiation, and/or chemotherapy and the only cure for MPNST is complete surgical resection [2]. Unfortunately, MPNSTs are often inoperable, due to nerve-association and infiltration of surrounding structures [3]. MPNSTs are also highly aggressive and often chemo- and radiation resistant, making these tumors difficult to treat [4]. Even when surgery in combination with radiation or chemotherapy is used, the local recurrence rate is 40-65%, with distant recurrence occurring 40-68% of the time. Chemotherapy treatment on distant metastases does not improve survival rates [5]. With the current treatment strategies used, the five-year survival rate for patients with MPNSTs is less than 40% and remains the leading cause of death for patients with NF1 [2, 6, 7]. Current research in the field focuses on identifying the genetic changes and molecular pathways that drive MPNST formation in order to identify targeted therapies that can be used to treat these tumors more effectively [3, 6].

In NF1 patients, loss of the *Neurofibromin 1* gene (*NF1*) is required for benign neurofibroma formation, and additional genetic changes are required for transformation into MPNSTs [8]. For example, in chimeric mice with loss of *Nf1,* benign, grade 1 neurofibromas will form; when *Tp53* is also lost, these tumors present as high-grade peripheral nerve sheath tumors (GEM-PNST) [9, 10]. When *Nf1* is biallelically inactivated in Schwann cells, GEM-grade 1 neurofibromas occur, but when *phosphatase and tensin homolog* (*Pten*) is also lost, these mice form numerous, high-grade PNSTs [11, 12]. Validating these genetically engineered mouse models (GEMMs), MPNSTs from human patients also often show reduced *PTEN* expression compared to normal nerve or benign neurofibromas, and alteration in TP53 [2, 12]. Less is known about the genetic changes that occur in spontaneous MPNSTs, but alterations in sporadic and NF1-associated MPNSTs include over-expression of the Epidermal Growth Factor Receptor (EGFR), loss of *PTEN, TP53* alteration, and loss of Cyclin-dependent kinase inhibitor 2A (*CDKN2A*) [2, 8, 13-15].

One common feature of sporadic and NF1 driven MPNSTs is activation of the MAPK and PI3K/AKT/mTOR pathways [4, 16, 17]. *NF1* encodes the protein Neurofibromin, a Ras GTPase activating protein (Ras-GAP) [18]. When *NF1* is lost, Ras accumulates in its GTP-bound, active state, resulting in hyperactive signaling through both the MAPK and PI3K pathway [18]. While loss of *NF1* results in increased signaling through the PI3K pathway, data suggest that additional changes occur that further activate signaling through this pathway. For example, many MPNSTs express EGFR, which when stimulated by Epidermal Growth Factor (EGF), results in hyperactivation of PI3K signaling [13]. Loss of *Nf1* in mouse Schwann cells is sufficient for benign neurofibroma formation, but additional loss of *Pten* drives malignant transformation [11, 12]. Additionally, a *Sleeping Beauty* forward genetic screen demonstrated that while benign neurofibromas had insertions in either *Nf1* or *Pten,* these mutations only co-occurred in MPNSTs [19]. These observations suggest that co-targeting the mTOR and MAPK pathways may be an effective treatment for MPNSTs.

Therapies that target PI3K/AKT/mTOR and MAPK pathways have been of studied both pre-clinically and in clinical trials for many cancer types [4, 20, 21]. The most clinically studied are drugs that bind FKBP12, inhibiting the mammalian target of rapamycin pathway by directly binding the mTOR Complex1 (mTORC1), not inhibiting kinase activity directly [22]. These inhibitors of the mTOR pathway include sirolimus (rapamycin) and its derivative, Everolimus (RAD001) [22]. Currently, Everolimus is FDA approved for kidney cancer, subependymal glial cell astrocytomas, pancreatic cancer and certain subtypes of breast cancer [23]. There are phase III clinical trials in progress for gastric cancer, hepatocellular carcinoma, and lymphoma [23].

Preclinical studies have been conducted in models of neurofibromas and MPNSTs using mTOR inhibitors that suggest varied efficacy at different stages of disease. In a mouse MPNST model where *Nf1* and *p53* are deleted in *cis*, and in MPNST xenograft studies, rapamycin treatment resulted in delayed tumor formation [24, 25]. In contrast, when *Nf1* is biallelically inactivated in Schwann cells and their precursors (*Nf1 ^flox/flox^; DhhCre*), modeling grade 1 neurofibromas, Everolimus was ineffective at decreasing tumor volume [26]. Based on these studies, phase II clinical trials are currently in progress using the mTOR inhibitor rapamycin in the treatment of plexiform neurofibromas [2].

PD0325901 (PD-901) is a potent and highly specific allosteric inhibitor of Mitogen-activated protein kinase kinase kinases, MEK1 and MEK2 (MEK) [27]. It is currently in clinical trials for non-small cell lung cancer and other advanced cancers [28]. In preclinical studies, PD-901 has shown efficacy in a human MPNST xenograft model; tumor growth was reduced and survival was prolonged, although tumor growth was not completely suppressed [29]. In GEMM-PNSTs in which Nf1 and Ink4a are biallelically deleted in the sciatic nerve, mice develop MPNSTs and show a delay in growth when treated with PD-901 [30]. In the GEMM of neurofibromas formation (*Nf1 ^flox/flox^; DhhCre*), PD-901 reduced tumor volume, but did not result in the induction of apoptosis of tumor cells [29].

To date, no clinical trials evaluating molecularly targeted therapies have prevented neurofibroma formation, stopped the growth of neurofibromas, or caused prolonged arrest of MPNST growth [31]. In preclinical studies using Everolimus or PD-901, it has been shown that, while these drugs can be either modestly or transiently effective, in most cases, each inhibitor acts cytostatically and does not induce apoptosis [32]. It is thought that this could be due to the effect of negative feedback, and/or the need to inhibit multiple pathways to elicit a cytotoxic response [20, 21]. In light of these findings, we sought to address whether co-targeting the mTOR and MAPK pathways would be more effective in treating MPNSTs than targeting either pathway alone. We addressed this by testing the effect of each of these drugs alone and in combination in two GEMM-PNSTs. One model represents NF1-associated MPNSTs, in which both *Nf1* and *Pten* are biallelically deleted in Schwann cells and their precursors (*Dhh-Cre; Nf1 ^flox/flox^; Pten ^flox/flox^*) [12]. The other model more closely resembles spontaneous MPNSTs in which *Pten* is biallelically inactivated in Schwann cells and *EGFR* is overexpressed (*Dhh-Cre; Pten ^flox/flox^; CNPase-hEGFR*) [13].

Here we confirm that targeting the MAPK pathway with the MEK inhibitor PD-901 and targeting the PI3K/AKT/mTOR pathway with the mTOR inhibitor Everolimus is effective at inhibiting cellular growth in both NF1-assscociated and spontaneous MPNST cell lines *in vitro,* and that these inhibitors appear to act cytostatically. When given in combination, Everolimus and PD-901 synergistically inhibit proliferation and effectively induce apoptosis in multiple MPNST cell lines. Based on this *in vitro* data, we tested the efficacy of these inhibitors as single agents and in combination in NF1-associated and spontaneous GEMM-PNST. While Everolimus and PD-901 alone each reduce tumor burden and/or grade, the combination of these two drugs is much more effective, resulting in a reduction in tumor burden, size and grade as well as an increase in survival in both mouse models. When given as single agents, these drugs are initially effective at reducing signaling through their respective pathways, but long term treatment results in the development of drug resistance, with reactivation of the target pathways. In contrast, when Everolimus and PD-901 are given simultaneously, signaling through both the PI3K and MAPK pathway remains effectively and persistently inhibited.

## RESULTS

### Everolimus and PD-901 are effective at inhibiting cellular growth in both NF1-associated and spontaneous MPNST cell lines

**Figure 1:**
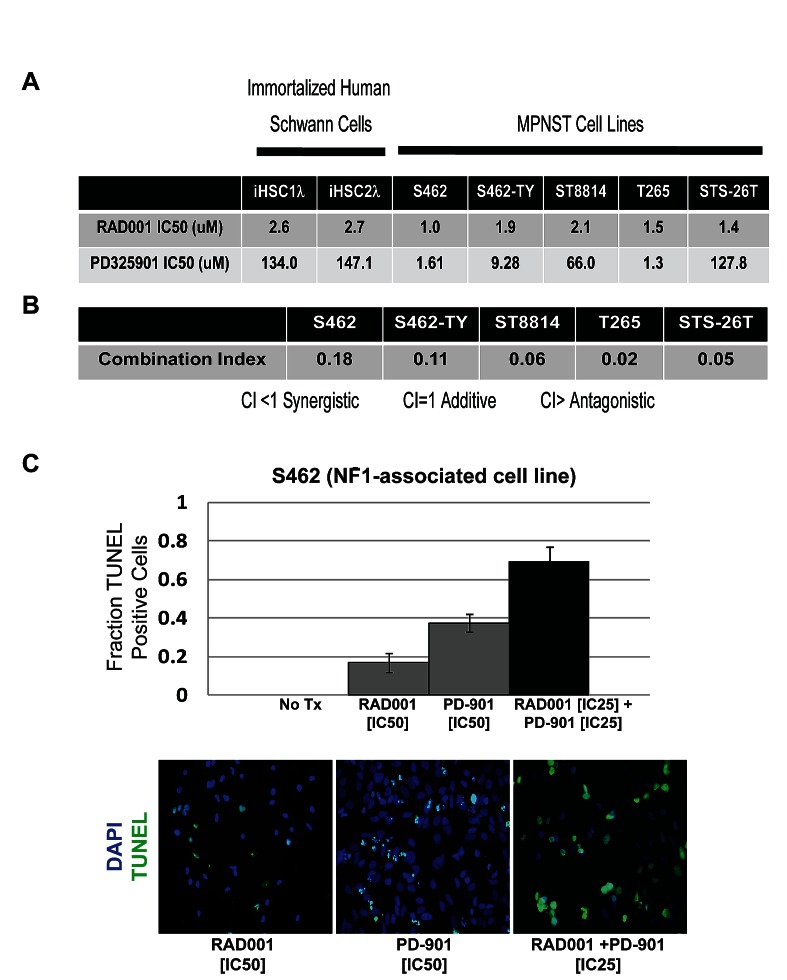
Everolimus (RAD001) and PD-901 are effective at inhibiting human MPNST cell growth and are synergistic at inhibiting cell growth and inducing apoptosis *in vitro* A. Inhibitory concentrations of 50% (IC_50_) for Everolimus and PD-901 in two immortalized human Schwann cell lines (iHSC1λ, iHSC2λ) and five MPNST cell lines (S462, S462-TY, ST8814, T265, and STS-26T) were determined using the MTS assay for proliferation and calculated using Calcusyn software ©. Immortalized Schwann cells are more resistant to inhibition of cell growth by Everolimus and PD-901, while the MPNST cell lines are much more sensitive as shown by their lower IC_50_ values. B. The combination index (CI) for Everolimus and PD-901 was determined using MTS cell growth data for the combination of drugs at varying IC_50_ dilutions and calculated using Calcusyn © software. Combination indices of less than one suggest that the two drugs act synergistically to inhibit cell growth in all five MPNST cell lines. C. TUNEL assay assessing apoptosis in the S462 MPNST cell line treated with IC_50_ doses of Everolimus or PD-901 as single agents. The percentage of cell undergoing apoptosis is less than 50%, suggesting that these drugs are acting cytostatically. In contrast, when Everolimus and PD-901 are given in combination at their IC25 doses, the percentage of apoptosis is nearly 70%, suggesting that in combination, these drugs act synergistically to induce apoptosis. Example images are shown of S462 cells stained with DAPI (blue) to allow total cell counts and TUNEL (green) to determine cells undergoing apoptosis. Error bars represent SEM.

To assess the therapeutic potential of inhibiting the PI3K/AKT/mTOR and MAPK pathways in human MPNSTs, a panel of two immortalized human Schwann cell lines (iHSC1λ and iHSC2λ [33]) and five human MPNST cell lines (S462 [34], S462-TY [35], ST8814 [36], T265 [37], and STS-26T [38]) were exposed to the mTOR inhibitor Everolimus and the MEK inhibitor PD-901. When exposed to Everolimus, immortalized Schwann cell lines were less sensitive to inhibition of cell growth than the MPNST cell lines, with 50% inhibitory concentrations (IC50s) of 2.6- 2.7 μM versus 1.0- 2.1 μM in the MPNST cell lines (Figure 1A). Similarly, PD-901 IC50s were 134.0μM- 147.1μM in the immortalized human Schwann cell lines versus 1.3- 127.8 μM in the MPNST cell lines (Figure 1A). These data suggest that MPNST cells are more sensitive to inhibition of mTOR and MEK than untransformed Schwann cells.

In addition to inhibiting cell growth at relatively low IC50s in MPNST cell lines as single agents, Everolimus and PD-901 also acted synergistically to inhibit cellular growth. MPNST cell lines treated with varying dilutions of Everolimus and PD-901 at their respective IC50s resulted in combination indices of 0.02 to 0.11 (Figure 1B). Combination indices below 1.0, as calculated using the software Calcusyn ©, suggest that in MPNST cell lines, Everolimus and PD-901 function to inhibit cellular growth synergistically.

### Inhibiting the mTOR and MEK in combination induces apoptosis in MPNST cell lines

To further understand how Everolimus and PD-901 function to inhibit cell growth synergistically, we exposed the S462 MPNST cell line to the IC50 doses of Everolimus and PD-901 and assessed apoptosis. In both cases, treatment with a single agent at a concentration that inhibits 50% cell survival resulted in less than 50% cell death, demonstrating that these drugs function largely cytostatically (Figure 1C). In contrast, when the S462 cells were treated with the IC25 doses of Everolimus and PD-901 in combination, the percentage of cells undergoing apoptosis was nearly 70%. These data suggest that co-targeting mTOR and MEK results in a synergistic induction of apoptosis.

### Everolimus and PD-901 as single agents are moderately effective at reducing disease in two GEMMs of MPNSTs

**Figure 2:**
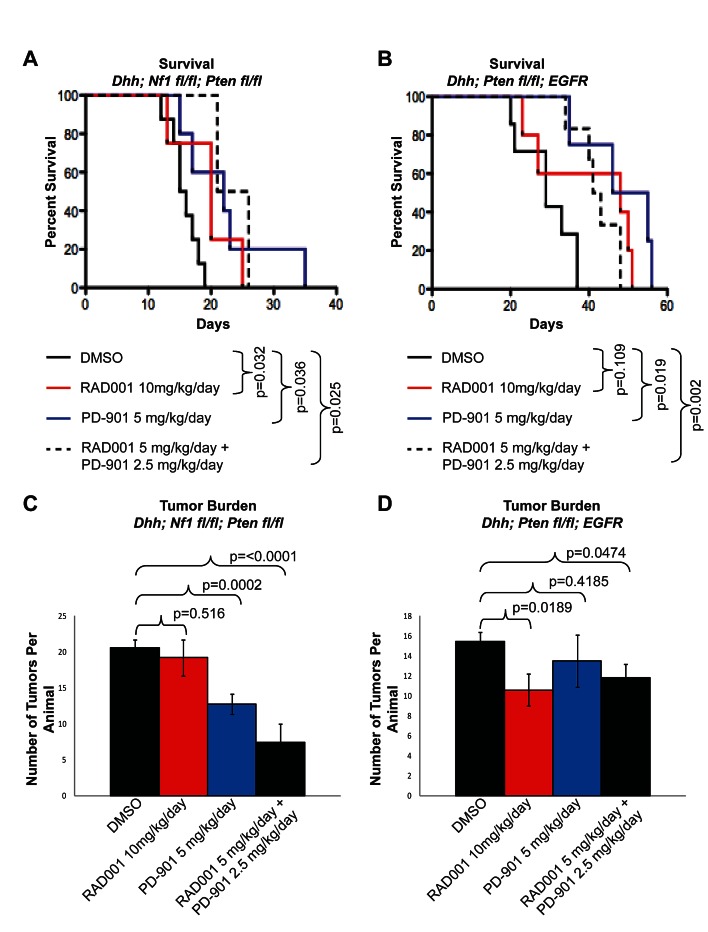
Everolimus (RAD001) and PD-901 alone and in combination can increase survival and reduce tumor burden in both the NF1-associated and spontaneous MPNST mouse models A. Kaplan-meier survival curve of the NF1-asociated MPNST mouse model. Treatment with Everolimus or PD-901 as single agents is able to extend lifespan as effectively as the combination therapy. B. In the spontaneous MPNST mouse model, Everolimus, PD-901, and the combination treatment were all able to extend the lifespan of mice. C. Tumor burden is reduced in the NF1-associated mouse model when treated with PD-901 or a combination of both Everolimus and PD-901. D. Tumor burden is reduced in the spontaneous MPNST mouse model when treated with Everolimus or a combination of both Everolimus and PD-901. Error bars represent SEM.

To test the efficacy of targeting the mTOR and MEK for MPNSTs *in vivo*, two GEMMs were treated with Everolimus and PD-901. In a mouse model of MPNSTs representing NF1-associated Schwann cell tumors, the *Desert Hedgehog* promoter was used to drive *Cre Recombinase* (*Dhh-Cre*) resulting in *Cre Recombinase* expression in Schwann cells and their precursors [39]. Combined with floxed alleles of *Nf1* (*Nf1 ^fl/fl^*) and *Pten* (*Pten ^fl/fl^*), expression of *Dhh-Cre* results in the biallelic inactivation of both *Nf1* and *Pten* in Schwann cells and their precursors [40, 41]. This GEMM has advantages for *in vivo* drug testing, including severe and fully penetrant peripheral nerve disease, a median lifespan of approximately 15 days, and an average tumor burden of 21.8 high-grade peripheral nerve sheath tumors (PNSTs) per animal [12]. Pups were divided into 4 cohorts and given DMSO (vehicle control), 10 mg/kg/day Everolimus, 5 mg/kg/day PD-901, or a combination of 5 mg/kg/day Everolimus and 2.5 mg/kg/day PD-901 beginning on post-natal day 0. When the NF1-associated MPNST mouse model (*Dhh; Nf1 ^fl/fl^; Pten ^fl/fl^*) was treated with Everolimus or PD-901, survival was significantly prolonged from an average of 15.8 days with DMSO treatment, to 22.4 days with Everolimus and 19.5 days with PD-901 (p=0.032 and p=0.036, respectively) (Figure 2A).

In a mouse model representing spontaneous MPNSTs, *Dhh-Cre* is again used to biallelically inactivate a floxed allele of *Pten,* and human *EGFR* is expressed under the control of the *2', 3' cyclic nucleotide 3/phosphodiesterase* promoter, which results in the over-expression of EGFR in Schwann cells [39, 41, 42]. In this model, mice have a median of 26 days, with 100% penetrance of high-grade PNSTs and approximately 13.7 tumors per animal [13]. Pups were separated into the same 4 cohorts described above for the NF1-associated mouse model and treatment also began on post-natal day 0. When the spontaneous MPNST mouse model (*Dhh; Pten ^fl/fl^; EGFR*) was treated with Everolimus or PD-901, survival was also extended from an average of 29.4 days with DMSO to 39.8 days with Everolimus and 48 days with PD-901 (p=0.109 and p=0.019, respectively) (Figure 2B).

Although Everolimus extended lifespan in the NF1-associated mouse model, the number of tumors per animal was not significantly reduced at the time of necropsy (Figure 2C). PD-901 treatment in the NF1-associated mouse model significantly reduced tumor burden from 20.6 tumors per animal with DMSO treatment to 12.8 tumors per animal with PD-901 treatment (p=0.0002) (Figure 2C). In contrast to the NF1-associated model, when the spontaneous mouse model was treated with Everolimus, the number of tumors per animal was reduced from 15.4 tumors per animal in the DMSO treated cohort, to 10.6 tumors per animals treated with Everolimus (p= 0.0189) (Figure 2D). PD-901 did not significantly reduce tumor burden in the spontaneous mouse model (Figure 2D).

**Figure 3:**
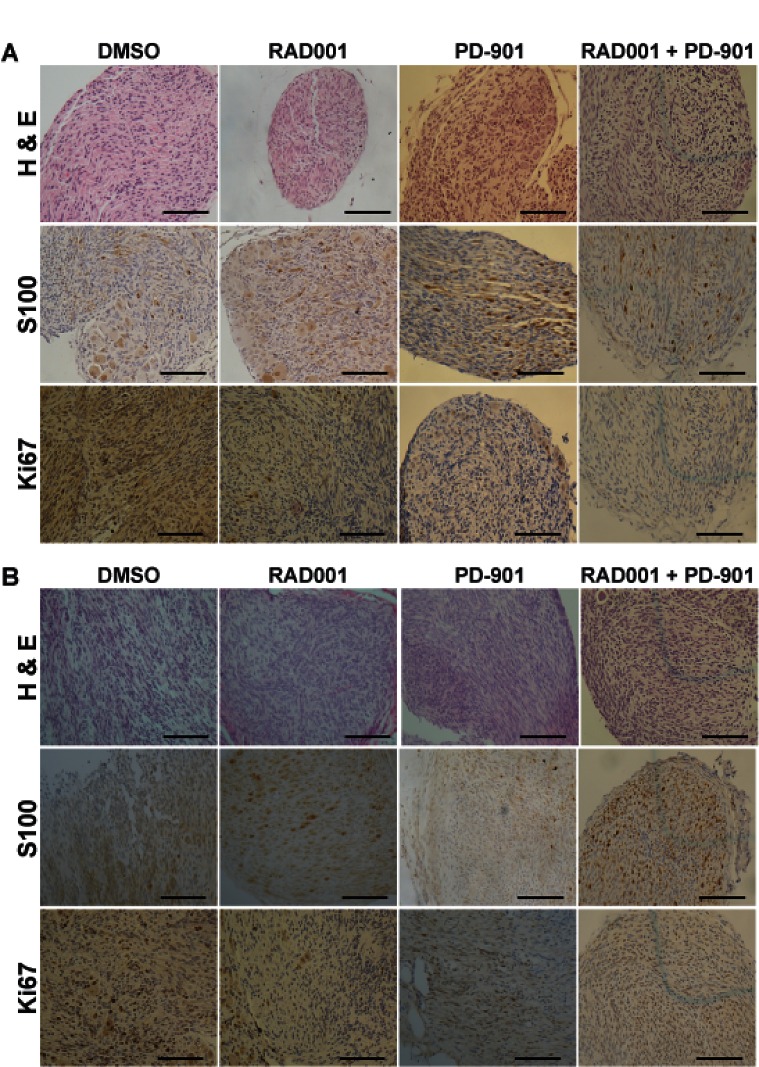
Immunohistochemical analysis of drug treated tumors A. Hematoxylin and eosin (H & E) staining was performed to assess cellularity and mitotic index, immunohistochemistry for S100β confirmed the Schwann cell origin of these tumors, and Ki67 immunohistochemistry demonstrated the level of cellular proliferation in *Dhh; Nf1 ^fl/fl^; Pten ^fl/fl^* mice treated with DMSO (vehical control), Everolimus, PD-901, and the combination treatment of Everolimus and PD-901. B. H & E, S100, and Ki67 analysis were performed for *Dhh; Pten ^fl/fl^; EGFR* mice treated with DMSO (vehical control), Everolimus, PD-901, and the combination treatment of Everolimus and PD-901. Scale bars represent 50 um.

**Figure 4:**
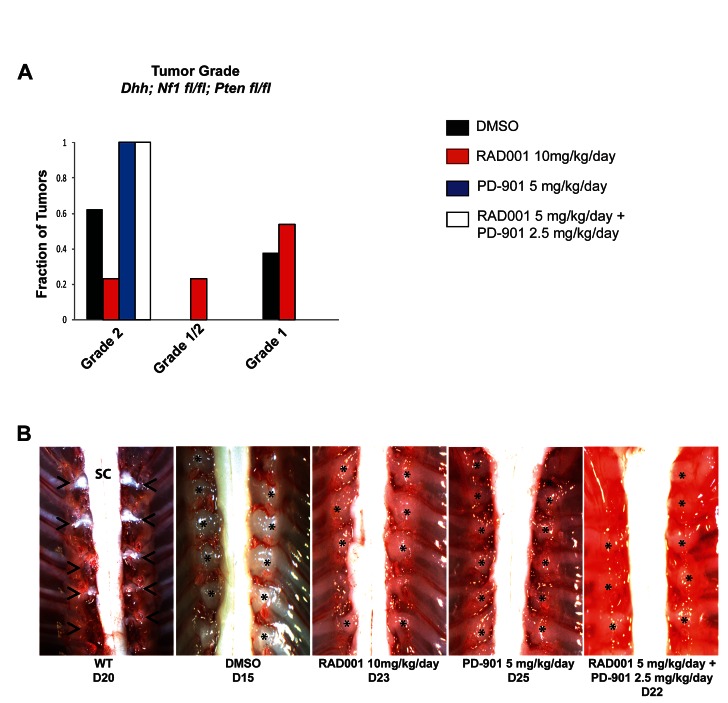
Tumor grade and size are reduced when the NF1-associated MPNST mouse model is treated with a combination of Everolimus (RAD001) and PD-901 A. The majority of tumors from mice treated with the vehicle control (DMSO) are grade 2, with the rest being grade 1. When mice were treated with Everolimus, tumor grade was reduced, with the majority of tumors being grade 1. When mice were treated with PD-901 or the combination of Everolimus and PD-901, despite having a reduced number of tumors, all tumors were grade 2. B. Example images of dorsal root ganglia (DRG) tumors in the NF1-associated mouse model with various treatments. DMSO treated mice have nearly every DRG highly enlarged, while Everolimus or PD-901 treatments reduce the size and number of enlarged DRGs. The combination treatment of Everolimus and PD-901 most effectively reduces the number and size of DRGs in the NF1-associated mouse model. Astrisks denotes DRG nerve; Arrow heads indicate tumors of the dorsal root ganglia; SC, spinal cord

Tumors treated with each drug were histologically analyzed to determine tumor grade as previously described [43, 44]. Hematoxylin and eosin (H & E) staining was performed to assess cellularity and mitotic index, immunohistochemistry for S100β confirmed the Schwann cell origin of these tumors, and Ki67 immunohistochemistry demonstrated the level of cellular proliferation (Figure 3A). Although the number of tumors per animal in the NF1-associated mouse model was not reduced, tumor grade was much lower with Everolimus treatment (Figure 3A & 4A). Tumors from mice treated with DMSO were mostly grade 2 PNSTs, with some being grade 1. When these mice were treated with Everolimus, there was a dramatic reduction in the fraction of grade 2 tumors, with most tumors being grade 1 following treatment. While PD-901 treatment alone dramatically reduced tumor burden in this model, all tumors analyzed were grade 2. Figure 3B & 4B show examples of the dorsal root ganglia (DRG) tumors in each of the treatment groups. DMSO treated mice have nearly every DRG nerve enlarged at only 15 days. In contrast, Everolimus and PD-901 treatment resulted in fewer and smaller tumors at later time points (day 23 and 25, respectively).

**Figure 5:**
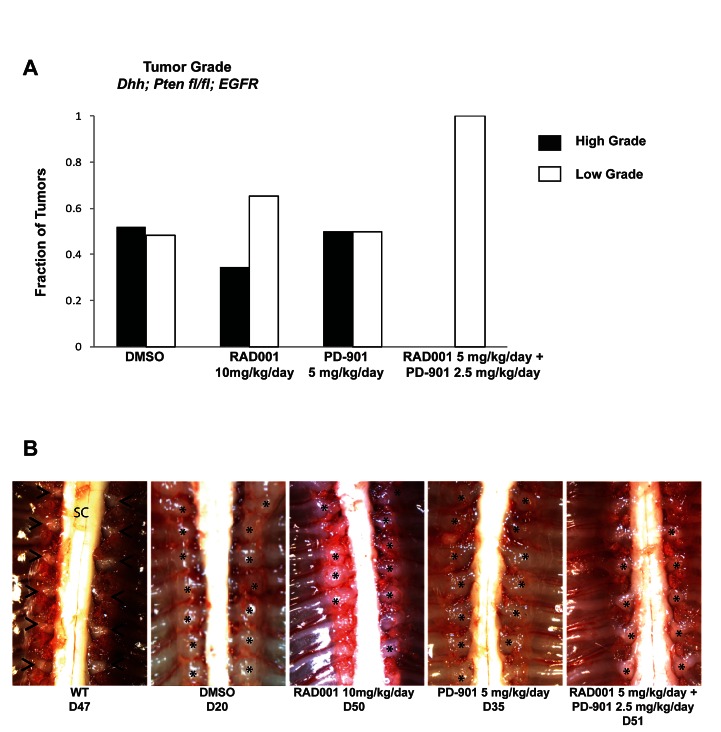
Tumor grade and size is reduced when the spontaneous MPNST mouse model is treated with Everolimus (RAD001) and PD-901 in combination A. Vehicle control (DMSO) treated mice have approximately 50% high grade tumors (grade 2/3 and 3) and 50% low grade tumors (grade 1-2). Treatment with Everolimus reduces the fraction high grade tumors and increases the fraction of lower grade 1 tumors, with some enlarged dorsal root ganglia were even categorized as nerve hyperplasia. Treatment with PD-901 did not change the fraction of high or low grade tumors. Treatment with the combination of Everolimus and PD-901 most dramatically reduced tumor grade resulting in all tumors being assessed as low grade. B. Example images of dorsal root ganglia (DRG) tumors in the spontaneous MPNST mouse model with various treatments. DMSO treated mice have nearly every DRG highly enlarged, while Everolimus or PD-901 treatments reduce the size and number of enlarged DRGs. The combination treatment of Everolimus and PD-901 most effectively reduces the size of DRGs in the spontaneous MPNST mouse model. Astrisks denotes DRG nerve; Arrow heads indicate tumors of the dorsal root ganglia; SC, spinal cord.

In the spontaneous MPNST mouse model, DMSO treated animals had approximately 50% low grade, neurofibroma-like tumors (grade 1-2) and 50% high grade (grade 2/3 and 3) tumors, as assessed by H & E, S100, and Ki67 as described above (Figure 3B & 5A). When these mice were treated with Everolimus, tumor grade was reduced, with the majority of tumors being low grade neurofibromas, and some enlarged DRGs even being graded as peripheral nerve hyperplasia. PD-901 was not effective at reducing tumor grade. Figure 5B shows examples of DRG tumors with each treatment. DMSO treated animals had nearly every DRG nerve enlarged, while mice treated with Everolimus and PD-901 had fewer and/or smaller DRG tumors, at later time points.

### Simultaneously inhibiting both mTOR and MEK in vivo is effective in both NF1-associated and spontaneous MPNST mouse models

Co-targeting mTOR and MEK in both mouse models resulted in a significant improvement in survival compared to vehicle control (DMSO) treated animals (23.5 days, p= 0.025 in the NF1-asociated model and 42.3 days, p= 0.002 in the spontaneous model), although the extension of lifespan was similar to treatment with Everolimus or PD-901 alone (Figure 2A-B). We found that treatment of mice with a combination of Everolimus and PD-901 at doses given as single agents (10 mg/kg/day and 5 mg/kg/day, respectively), resulted in toxicity, with mice becoming very slow and shakey in their movements, and showing signs of intestinal damage. To avoid toxicity, mice were treated with significantly lower doses of drug in combination (5 mg/kg/day Everolimus and 2.5 mg/kg/day PD-901). Both mouse models had significant reductions in tumor burden when drugs were combined (7.5 tumors per animal in the NF1-asociated model, p <0.0001 and 11.8 tumors per animal in the spontaneous model, p=0.0474) (Figure 2C-D).

Treatment with Everolimus and PD-901 in combination in the NF1-asssociated mouse model extended lifespan and dramatically reduced tumor burden, although all tumors that were analyzed at necropsy were grade 2, as assessed by H & E, S100, and Ki67 as described above (Figure 2A, 2C, 3A, 4A). Importantly, the DRG tumors from mice treated with the combination therapy were somewhat smaller at necropsy (Figure 4B). In contrast, combination treatment of Everolimus and PD-901 in the spontaneous mouse model resulted in all low grade tumors (Figure 3B, 5A). Additionally, fewer enlarged DRG were observed, and those seen in this model were quite small (Figure 5B).

### Inhibiting mTOR or MEK signaling results in the development of drug resistance with reactivation of signaling through each respective pathway

**Figure 6:**
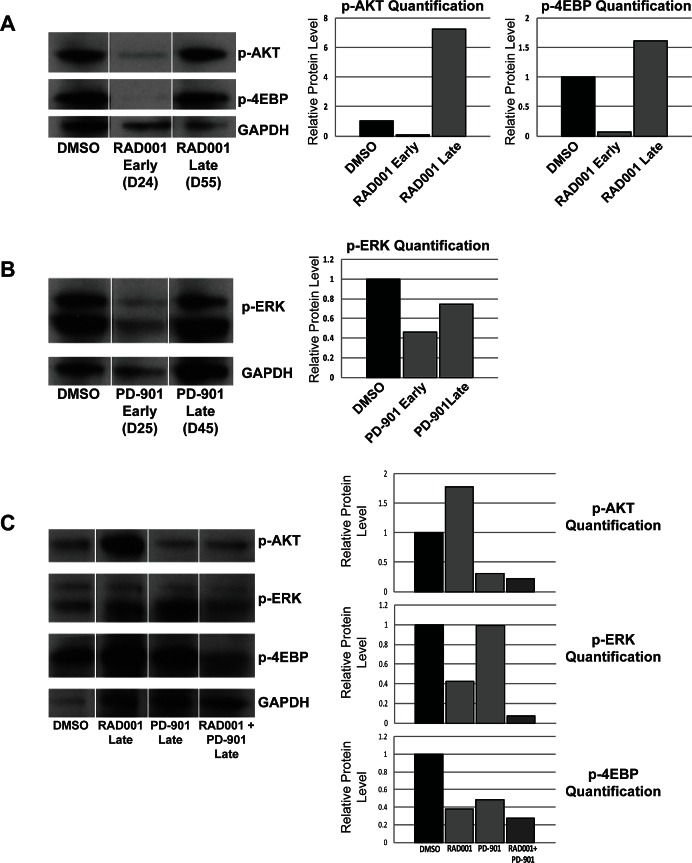
Treatment with Everolimus (RAD001) or PD-901 as single agents is effective at early time points, but resistance develops at later time points, while treating mice with a combination of Everolimus and PD-901 results in persistent and prolonged inhibition of signaling through the MAPK and PI3K pathways A. Western blot analysis and quantification of phospho-AKT and phospho-4EBP in DRG tumors from animals treated with DMSO or Everolimus at early and later time points. At early time points, Everolimus effectively reduces phospho-AKT levels, but at later time points, the level of phospho-AKT is very high. Similarly, at early time points, Everolimus also effectively reduces phospho-4EBP levels, but at later time points, the level of phospho-4EBP is very high, suggesting the development of resistance to Everolimus treatment. B. Western blot analysis and quantification of phospho-ERK levels in DRG tumors from animals treated with DMSO or PD-901 at early and later time points. At early time points, phospho-ERK is inhibited, but at later time points the level of phospho-ERK increases suggesting that these mice are becoming resistant to PD-901 treatment. C. Western blot analysis of DRG tumors treated with DMSO, Everolimus, PD-901, or a combination of Everolimus and PD-901 at late time points. Quantification of western blot demonstrates that at late time points, the level of phospho-AKT is elevated in Everolimus treated animals, but remains low when animals are treated with a combination of Everolimus and PD-901. Similarly, the level of phospho-4EBP remains lowest when mice are given the combination treatment. Phosho-ERK levels also remain lowest when mice are given Everolimus and PD-901 in combination.

To assess pathway inhibition *in vivo,* we monitored the level of p-AKT, p-4EBP, and p-ERK in tumors from animals treated with Everolimus or PD-901. As expected, in mice treated with Everolimus, the levels of p-AKT and p-4EBP were reduced at early time points (day 24) at which there were few tumors, all of which were relatively small (Figure 6A). At later time points (day 55), tumors from Everolimus treated mice were more numerous and larger, and western blot analysis showed that both p-AKT and p-4EBP were elevated, suggesting that long term treatment of Everolimus results in the development of drug resistance (Figure 6A). Similarly, tumors from mice treated with PD-901 at early time points (day 25) have a reduced level of p-ERK (Figure 6B). At later time points (day 45), the level of p-ERK has strongly increased, again suggesting that long term PD-901 treatment results in the development of resistance (Figure 6B).

### Co-targeting mTOR and MEK results in prolonged and persistent inhibition of signaling through both pathways

Because long term treatment with Everolimus or PD-901 resulted in reactivation of the PI3K/AKT/mTOR and MAPK pathways, we sought to understand the mechanism by which the combination therapy more effectively treats disease in our models. In contrast to the reactivation of the MEK and mTOR signaling seen in single drug treatments, when mice were treated with Everolimus and PD-901 in combination, p-AKT, p-4EBP, and p-ERK were all inhibited at early timepoints, and remained low at later time points (Figure 6C). This suggests that co-targeting the mTOR and MEK pathways *in vivo* results in prolonged and persistent pathway inhibition, resulting in an effective combinational targeted therapy.

### DISCUSSION

Analysis of human Schwann cell tumors and mouse models of neurofibromas and MPNSTs have demonstrated the functional importance of both PI3K/AKT/mTOR and MAPK pathway activation [17, 18, 29, 30]. While targeting either of these pathways alone in preclinical models of MPNST has shown moderate efficacy, we show that co-targeting mTOR and MEK is more effective both *in vitro* and *in vivo* than targeting either pathway alone. In MPNST cell lines, we show that a combination of the mTOR inhibitor, Everolimus, and the MEK inhibitor, PD-901, act synergistically to inhibit growth and induce cell death (Figure 1). While many targeted therapies, including these, have been shown to act cytostatically, it is of clinical importance that, in combination, these inhibitors act cytotoxically [20, 21].

We believe that this study has many advantages over previous pre-clinical studies. First, GEMMs better reflect patient outcomes than *in vitro* and xenograft studies, as these tumors model the genetic changes, progressive evolution, anatomic location, pathology, histology, and micro-environmental interactions of human tumors [45]. In addition, as these tumors form *in situ,* the contribution of other cell types can be evaluated [45]. This is especially important in studying Schwann cell tumors, as these tumors are quite angiogenic, contain heterogeneous populations of fibroblasts, macrophages, and mast cells, and are highly associated with peripheral nerves [1, 46]. Another advantage of our models is that these mice rapidly and uniformly form high-grade PNSTs [12, 13]. While other models have evaluated the pre-clinical efficacy of targeted therapies in neurofibromas, it is important to evaluate these therapies in mouse models of PNSTs, as clinical trials are likely to evaluate these therapies in malignant tumors.

Another important aspect of this preclinical study is that targeting the PI3K/AKT/mTOR and MAPK pathways alone and in combination was studied in two preclinical mouse models that represent two subsets of Schwann cell tumors, those that form in the context of NF1 and those that form spontaneously [12, 13]. By showing efficacy of these targeted therapies in two different GEMMs, we hope that there will be an increased likelihood of success in human clinical trials. Finally, these GEMMs have advantages for pre-clinical drug testing, as the mice rapidly form tumors with 100% penetrance and multiple tumors per animal [12, 13].

We show that survival is significantly increased in both mouse models when the mice are treated with Everolimus, PD-901, or the combination of both (Figure 2). In the NF1-associated mouse model, the combination of Everolimus and PD-901 significantly decreased tumor burden and size, although tumor grade remained high (Figure 2-4). In the spontaneous MPNST mouse model, tumor burden, size, and grade were all significantly decreased when Everolimus and PD-901 were administered in combination (Figure 2, 3 & 5). While these drugs clearly demonstrated efficacy in these models, we have not ruled out the possibility that in addition to inhibiting the malignant Schwann cells, these drugs could also be affecting non-neoplastic cells, such as mast cells and fibroblasts, and may be having an effect on angiogenesis and tumor vasculature. Finally, it should be noted that PD-901 has been shown to have off target effects [47]. Although the off target effects of this inhibitor may be playing a role in our study, we have demonstrated that PD-0325901 does in fact inhibit MEK (Figure 6), and the use of other MEK inhibitors would be necessary to determine whether specifically inhibiting MEK is necessary for the effect seen in our study. Although there may be off target effects, PD-901 is still being used in preclinical studies, as well as multiple clinical trials for cancers dependent on MAPK signaling.

Further research will be necessary to determine the exact mechanism by which PI3K/AKT/mTOR and MAPK signaling cascades become activated in benign Schwann cell tumors and MPNSTs. While loss of *NF1* is clearly functioning to drive benign neurofibromas, it appears that further activation of Ras/PI3K/AKT/mTOR signaling is required for malignant transformation [1, 8-10]. We have previously showed a reduction in *PTEN* expression in both human and murine MPNSTs, but a mechanistic understanding of how *PTEN* expression is down-regulated may uncover other potential drug targets, such as miRNAs and control of methylation [12, 48]. Clearly, in Neurofibromatosis Type 2 (NF2), these mechanisms play a role in Schwann cell tumorigenesis, and have been utilized in preclinical studies as promising drug targets [49, 50]. Another recent study reported the identification of FAM83B as a novel activator of PI3K/AKT/mTOR and MAPK signaling that was capable of driving transformation of human mammary epithelial cells [51]. This study also implicates FAM83B as a novel target for drug therapy, and further work should be pursued to see whether FAM83B plays a role in Schwann cell tumors as well [51].

The development of resistance to targeted therapies in cancer has been well documented [21, 52]. We show that when mice are treated with either Everolimus or PD-901 as single agents over a long period of time, resistance does indeed develop, and the PI3K/AKT/mTOR and MAPK pathways become reactivated (Figure 6). In contrast, when mTOR and MEK are co-targeted in these two mouse models, both signaling pathways remain inhibited over the course of treatment (Figure 6). This likely accounts for the reason that single drug treatments do not show the efficacy that the combination therapy shows in these two mouse models and may explain why either of these drugs given alone in other models have only showed modest efficacy [25, 26, 29]. It is also likely that mice treated with Everolimus and PD-901 in combination may also develop resistance, although more delayed than with single treatments, through different mechanisms, as these mice eventually succumb to their tumors due to paralysis. Future work will be required to determine if other pathways become activated, allowing for tumor cells to escape inhibition from Everolimus and PD-901.

Future work will also need to be done to study the effect of co-targeting mTOR and MEK in other less severe models of neurofibromas or MPNST development, including other GEMMs as well as xenograft models. In fact, in slower developing tumor models, these drugs may be more effective, as they have more time to effectively inhibit these pathways and control cellular proliferation and transformation. It will be important to determine the safety and efficacy of co-targeting the mTOR and MEK pathways in human patients. We found that treatment of mice with a combination of Everolimus and PD-901 at doses given as single agents resulted in toxicity. To avoid toxicity, mice were treated with significantly lower doses of drug in combination, and this approach may be necessary in clinical trials as well. It is also of interest to study whether the combination of Everolimus and PD-901 could be used to treat plexiform neurofibromas in an attempt to prevent transformation to MPNSTs. This could be done in the *Dhh-Cre; Nf1 ^flox/flox^* model [11]. One difficulty that could arise that we and others have demonstrated, is that long term treatment with Everolimus causes immunosuppresion, which could add a layer of complexity to the use of this drug in a clinical setting over a long period of time for plexiform neurofibromas [53]. Additionally, it has become clear that the immune system can play an important role in cancer, therefore, immune system suppression may not be desirable [54]. While there are challenges with implementing new targeted therapies in the clinic, the data presented here demonstrates that co-targeting PI3K/AKT/mTOR and MAPK may be beneficial for patients suffering from Schwann cell tumors.

## MATERIALS AND METHODS

### Tissue Culture Reagents and Cell Lines

Cultured immortalized Schwann cells (iHSC1λ and iHSC2λ) were both derived from a patient's normal sciatic nerve, are *NF1* wild-type, and were immortalized by *hTERT* and *CDK4*^R24C^ to allow *in vitro* studies [33]. Immortalized human Schwann cell and MPNST cell lines (S462 [34], S462-TY [35], ST8814 [36], T265 [37], and STS-26T [38]) were maintained in Dulbecco's Modification of Eagle Medium (DMEM) supplemented with 10% fetal bovine serum and penicillin/streptomycin (Cellgro) and cultured on tissue culture-treated plates under standard conditions of 37°C and 5% CO2.

### *In Vitro* Drug Studies to Determine 50% Inhibitory Concentration (IC50) and Combination Index (CI)

Everolimus and PD-901 were solubilized in DMSO and then subsequently diluted in sterile PBS. 1,200 cells per well of a 96-well plate were treated with varying concentration of drug in quadruplicate and assayed for cell viability using the MTS assay (Promega) to determine the IC50 values, 48 hours after drug treatment. Drug was added at the time of cell plating. All data analysis was done using Calcusyn software (CalcuSyn Version 2.1, BioSoft). A combination of Everolimus and PD-901 at varying dilutions of their IC50 concentration was used to determine the combination index (CI) using Calcusyn software (CalcuSyn © Version 2.1, BioSoft).

### Generation of Transgenic Animals and PCR Genotyping

Generation of transgenic animals and PCR genotyping were done as previously described [12, 13].

### *In Vivo* Drug Studies

Everolimus and PD-901 were solubilized in DMSO and then subsequently diluted in PBS and sterilized. Mice were weighed daily and given 10 mg/kg/day of Everolimus or 5 mg/kg/day of PD-901. For combination studies mice were given 5 mg/kg/day of Everolimus and 2.5 mg/kg/day of PD-901 at the same time. Mice were weighed and monitored daily, and sacrificed when they became moribund. Tumors, nerves, and organs were harvested for immunohistochemistry and western blot analysis. All animal work was conducted according to the University of Minnesota's approved animal welfare protocol.

### Peripheral Nerve Tumor Analysis

Tumors were carefully removed from the sacrificed animal under a dissecting microscope (Leica), washed, and placed in cold phosphate buffered saline (PBS). Sciatic nerves, brachial plexi, sacral plexi, trigeminal nerves, spinal cord, brain, and liver were also removed and observed for any abnormalities. The number of enlarged dorsal root ganglia was counted for the whole spinal cord. All tumor nodules (>1 mm in diameter) were carefully removed from the spinal cord.

### Hematoxylin and eosin staining

Tissues were fixed in 10% formalin, routinely processed, and embedded in paraffin. Sections for histology were cut at five microns from the paraffin blocks using a standard microtome (Leica), mounted and heat fixed onto glass slides. Slides were stained with hematoxylin and eosin (H&E) using standard protocols.

### Mouse Tumor Immunohistochemistry (IHC)

Immunohistochemistry was done as previously described [12, 13]. Formalin fixed, paraffin embedded tissues were sectioned at five microns, mounted, and heat-fixed onto glass slides to be used for IHC analyses. The glass section slides were de-waxed and rehydrated through a gradual decrease in ethanol concentration. The antigen epitopes on the tissue sections were then unmasked using a commercially available unmasking solution (Vector Laboratories) according to the manufacturer's instructions. The tissue section slides were then treated with 3% hydrogen peroxide to remove endogenous peroxidases. Blocking was performed at room temperature in normal goat serum (5% serum in PBS) in a humidified chamber for one hour. Sections were then incubated overnight at 4°C in a humidified chamber with primary antibody (S100 (Santa Cruz 1:100), Ki67 (Novocastra 1:200)). After primary incubation, sections were washed thoroughly in PBS before incubating with goat anti-rabbit horseradish peroxidase conjugated-secondary antibody (Santa Cruz Biotechnology). After three washes with PBS, the sections were treated with freshly prepared DAB substrate (Vector Laboratories) and allowed to develop before stopping the reaction in water once adequate signal was obtained. Finally, sections were then lightly counter-stained with hematoxylin, dehydrated through gradual increase in ethanol concentration, cleared in Xylene (Fisher Scientific) and mounted in Permount (Fisher Scientific).

### Histologic Evaluation

Sections stained with H&E, Ki67 and S100β were evaluated for all tumors. Each sample was graded using established criteria for tumors arising in genetically engineered mice [43, 44]. Briefly, low-grade PNSTs exhibited low cellularity with little if any nuclear atypia and mitotic activity. High-grade PNSTs were increasingly cellular with increasing nuclear atypia and increasing mitotic activity.

### Immunofluorescence and TUNEL staining

For immunofluorescence assays, cells were grown to 80% confluency on 8 chambered slides (Lab-TekII). Cells were fixed in 10% formalin and washed with phosphate-buffered saline (PBS) with 0.1% Tween-20 (PBST). TUNEL staining was performed using the In Situ Cell Death Detection Kit, POD (Roche). Slides were mounted using Prolong Gold Antifade Reagent with DAPI (Invitrogen) and images using a Zeiss Axiovert 25 inverted microscope. For analysis of cell death, total cells and TUNEL positive cells were counted and averaged over three independent frames with 50-100 cells per frame.

### Western Blot Analysis

One million cells were lysed using an NP-40 buffer (50mM Tris-HCl pH 7.6, 150mM NaCl, 1% NP-40, 5mM NaF, 1mM EDTA) containing a protease inhibitor (Roche) and phosphatase inhibitors (Sigma). Whole cell lysates were cleared by centrifugation. Protein samples were prepared in an SDS solution with reducing agent (Invitrogen) and run on 10% Bis-Tris pre-made gels (NuPage, Invitrogen). Gels were transferred onto PVDF membranes using the iBlot system (Invitrogen) and activated in 100% methanol. Membranes were blocked in filtered 5% Bovine Serum Albumin (BSA) for 2 hours at room temp followed by a 4°C overnight incubation in primary antibody. The primary antibodies used in this study were: p-AKT S473 (Cell Signaling 1:100), p-4EBP (Cell Signaling 1:500), p-ERK (Cell Signaling 1:1000), and GAPDH (Cell Signaling 1:2000). Following primary antibody incubation, membranes were thoroughly washed in Tris Buffered Saline (TBS) with 0.1% Tween-20 (TBST) and incubated in goat anti-rabbit IgG-HRP conjugated secondary antibody (Santa Cruz, 1:4000 in 0.5% BSA, 1 hour at room temperature). Blots were thoroughly washed in TBST and developed using the SuperSignal WestPico Chemiluminescence Detection Kit (Thermo Scientific). Densitometry quantification was done using ImageJ software and normalized to GAPDH [55].
