# Correction: Co-targeting the MAPK and PI3K/AKT/mTOR pathways in two genetically engineered mouse models of schwann cell tumors reduces tumor grade and multiplicity

**DOI:** 10.18632/oncotarget.27349

**Published:** 2020-09-29

**Authors:** Adrienne L. Watson, Bryant J. Keller, Kyle A. Williams, Namrata N. Damle, Samuel J. Finnerty, Leah K. Anderson, Andrew D. Greeley, Vincent W. Keng, Eric P. Rahrmann, Amanda L. Halfond, Natasha M. Powell, Margaret H. Collins, Tilat Rizvi, Christopher L. Moertel, Nancy Ratner, David A. Largaespada

**Affiliations:** ^1^ Masonic Cancer Center, University of Minnesota, Minneapolis, MN, USA; ^2^ Department of Genetics, Cell Biology and Development, University of Minnesota, Minneapolis, MN, USA; ^3^ Center for Genome Engineering, University of Minnesota, Minneapolis, MN, USA; ^4^ Brain Tumor Program, University of Minnesota, Minneapolis, MN, USA; ^5^ Health and Natural Sciences Department, University of Minnesota, Minneapolis, MN, USA; ^6^ Department of Pediatrics, University of Minnesota, Minneapolis, MN, USA; ^7^ Division of Experimental Hematology and Cancer Biology, Cincinnati Children’s Hospital Medical Center, Cincinnati, OH, USA; ^8^ Division of Pathology and Laboratory Medicine, Cincinnati Children’s Hospital Medical Center, Cincinnati, OH, USA; ^9^ Department of Pediatrics, Cincinnati Children’s Hospital Medical Center, Cincinnati, OH, USA; ^10^ Department of Applied Biology and Chemical Technology, The Hong Kong Polytechnic University, Hung Hom, Kowloon, Hong Kong

**This article has been corrected:** After carefully repeating certain experiments and reviewing old research records, we’ve uncovered evidence that PD0325901 and RAD001 doses used for *in vivo* experiments, in Watson et al., 2014, were lower than intended. We found that in contrast to the original paper, Everolimus (Selleckchem S1120) and PD0325901 (Selleckchem S1036) were only tolerated in young mice at much lower concentrations than originally reported. Likely due to dilution errors, the mice in the original draft received significantly reduced amounts of drug. New dose escalation studies showed that the mice could tolerate 5mg/kg RAD001 thrice weekly and 0.5mg/kg PD0325901 thrice weekly as monotherapies or in combination. As monotherapies, both RAD001 and PD0325901 slightly extended the survival of genetically engineered mice with peripheral nerve sheath tumors (Dhh-Cre; Pten fl/fl ; Nf1 fl/fl ). Ten control mice were injected with DMSO and PBS vehicles. As seen in Figure 1, these animals succumbed to tumor burden at 18-19 days of age without treatment. When PD901 was administered in 6 animals, the median survival increased from 18 to 24 days. Similarly, and more profoundly, treatment with RAD001 increased the median longevity from 18 to 54 days. When the two drugs were used in combination, the median survival increased to 79 days. The combination treatment significantly improved longevity versus control and PD0325901 monotherapy. This trend towards improved longevity is very impressive considering that the untreated mice live only 18 days. Overall, these data agree with what was published originally in Watson et al., but show a stronger response, and suggest that these drugs could profoundly improve the prognosis of patients with rapidly growing or malignant peripheral nerve sheath tumors. Pharmacodynamic analyses of peripheral nerve sheath trigeminal nerve tumors in these mice show that increasing the dose of the MEK inhibitor PD0325901 leads to decreasing levels of phospho-ERK, coinciding with an apparent increase in phospho-AKT (Figure 2). This agrees with what was published in the initial Watson et al. paper. Thus, PD0325901 can suppress its target enzyme MEK, and concomitant AKT activation suggests a mechanism of resistance to monotherapy with this drug. Altogether, our new analyses verify what was found in Watson et al, including the in vitro studies (data not shown), although the *in vivo* effects of PD0325901, RAD001 and combined treatment are much stronger than first reported. Overall, the conclusions and trends drawn from the original paper have not changed substantially. That being said, when the correct doses were given, the *in vivo* data was much more robust and displayed an extraordinary increase in longevity. Our in vitro data verified what had been previously seen in pharmacodynamics in the Watson et al paper, showing dose dependent decreases in phosphorylated ERK with corresponding increases in phosphorylated AKT. Together these data draw much more impactful and profound conclusions while still holding true to the originally published trends.

**Figure 1:**
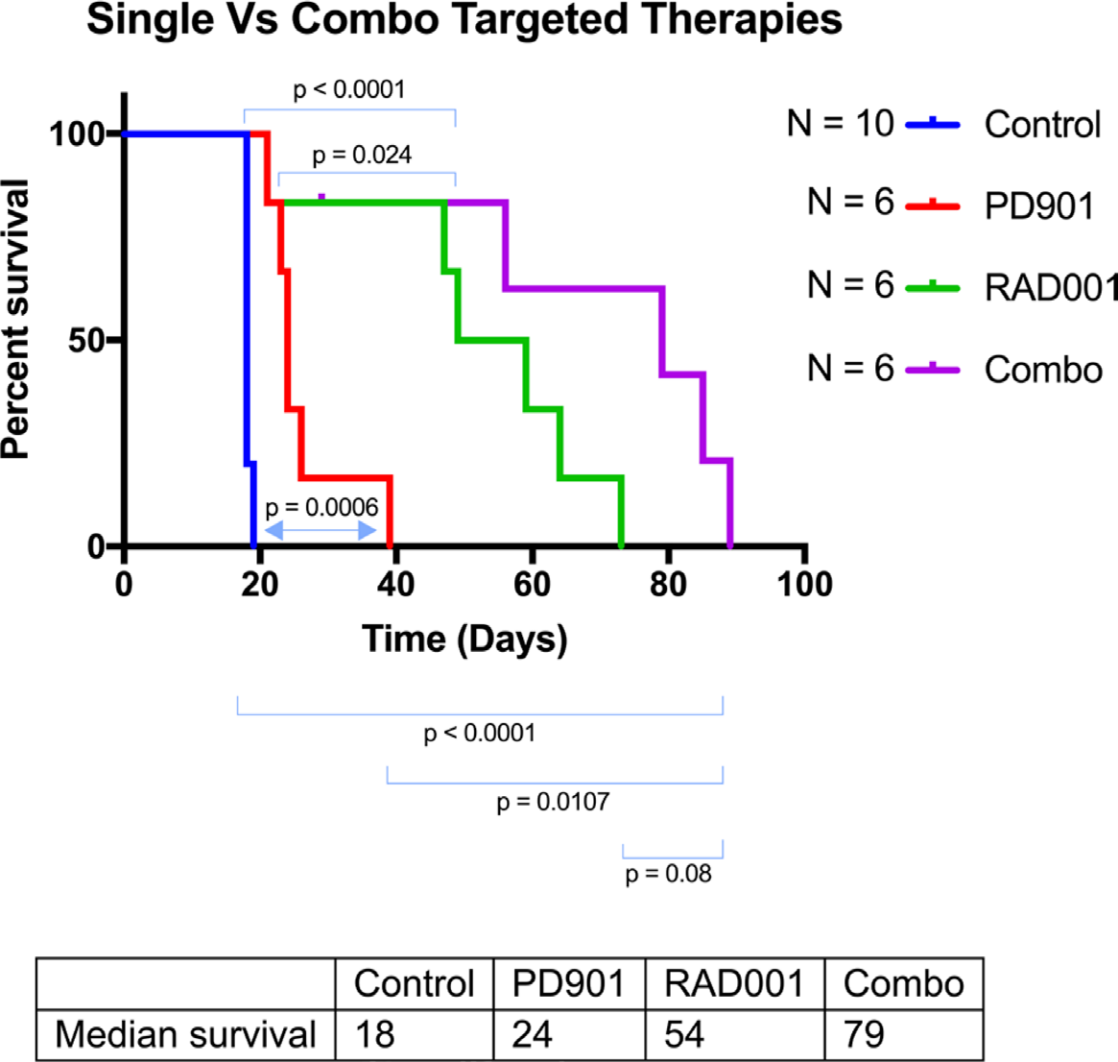
Kaplan-Meyer of the different treatment cohorts of the Genetically Engineered Peripheral Nerve Sheath Tumor mice. These *Dhh*-*Cre*; *Pten^fl/fl^*; *Nf1^fl/fl^* mice were injected with a PBS control IP, PD0325901 (0.5mg/kg IP thrice weekly), RAD001 (5mg/kg thrice weekly IP), or combination PD032590/RAD001. Control group mice displayed a median survival of 18 days. PD901 treatment modestly improved this to 24 days with a p value of 0.0006. RAD001 treatment improved this response even more profoundly, increasing the longevity to a median of 54 days with a p value of <0.0001. Finally, combination treatment showed an additive effect, increasing survival to 79 days (p<0.0001).

**Figure 2:**
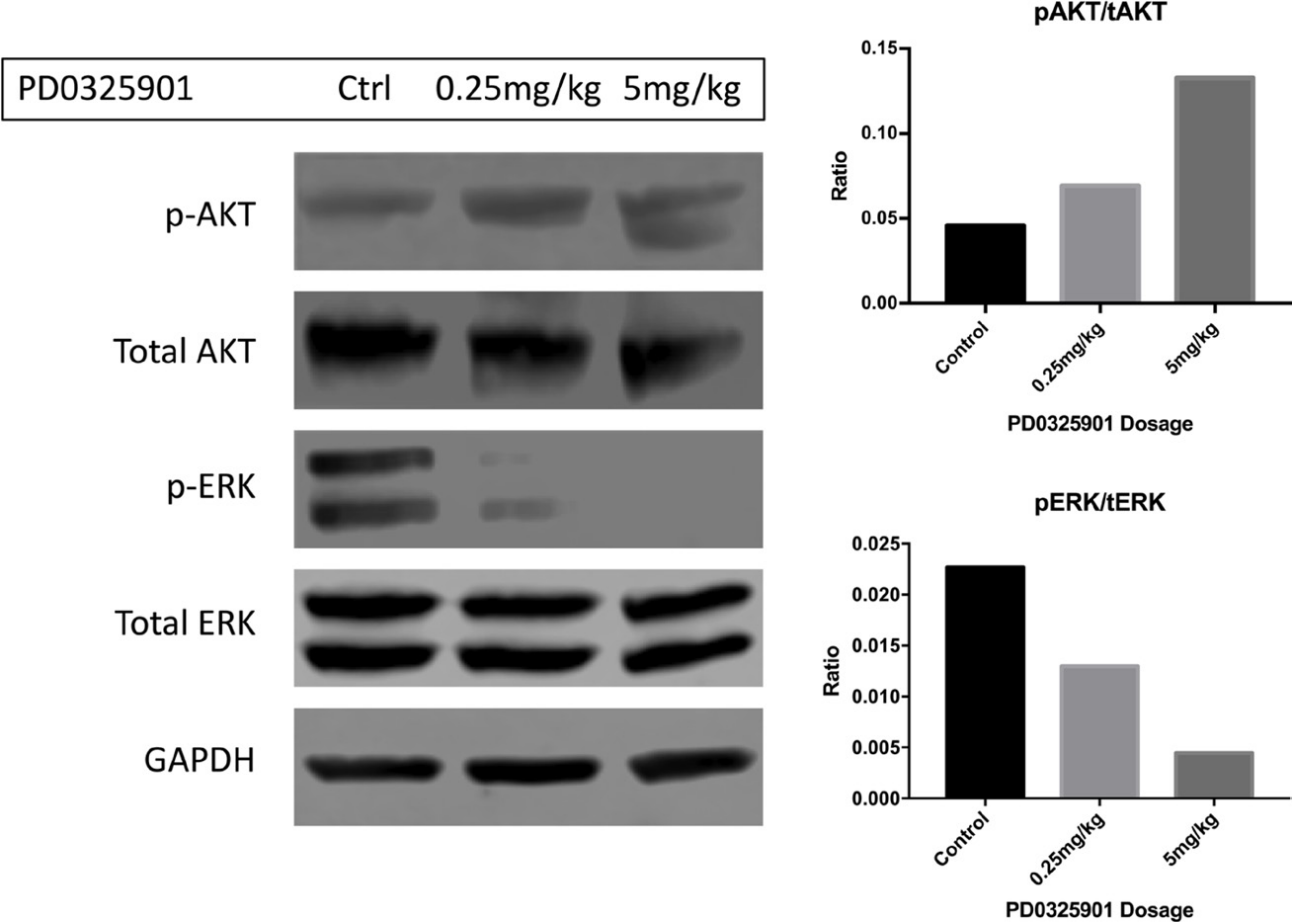
Phosphorylated ERK is diminished in response to increasing dosing of PD0325901 in the trigeminal nerves of *Dhh*-*Cre*; *Pten^fl/fl^*; *Nf1^fl/fl^* mice. In this western blot, activated ERK is diminished when PD901 was administered IP acutely 2 hours before sacrifice (Cell Signaling 4377S). At 5mg/kg, signal was ablated completely. In a contrasting manner, p-AKT (Cell Signaling 4075S) levels seemingly increased in the trigeminal nerves in a dose-dependent fashion. This correlates with a potential signaling feedback and resistance mechanism of monotherapeutic targeted treatment. Quantifications for phospho-AKT/total-AKT and phospho-ERK/total-ERK are pictured on the right panel.

In addition, the author list for this paper has been updated. The revised author list is shown below:

Adrienne L. Watson^1,2,3,4,*^, Bryant J. Keller^1,2,3,4,*^, Kyle A. Williams^1,2,3,4^, Namrata N. Damle^1,2,3,4^, Samuel J. Finnerty^1,2,3,4^, Leah K. Anderson^1,2^, Andrew D. Greeley^1,2^, Vincent W. Keng^1,2,3,4,10^, Eric P. Rahrmann^1,2,3,4^, Amanda L. Halfond^5^, Natasha M. Powell^4^, Margaret H. Collins^8^, Tilat Rizvi^8^, Christopher L. Moertel^6^, Nancy Ratner^7,9^, and David A. Largaespada^1,2,3,4,6^

^1^Masonic Cancer Center, University of Minnesota, Minneapolis, MN, USA

^2^Department of Genetics, Cell Biology and Development, University of Minnesota, Minneapolis, MN, USA

^3^Center for Genome Engineering, University of Minnesota, Minneapolis, MN, USA

^4^Brain Tumor Program, University of Minnesota, Minneapolis, MN, USA

^5^Health and Natural Sciences Department, University of Minnesota, Minneapolis, MN, USA

^6^Department of Pediatrics, University of Minnesota, Minneapolis, MN, USA

^7^Division of Experimental Hematology and Cancer Biology, Cincinnati Children’s Hospital Medical Center, Cincinnati, OH, USA.

^8^Division of Pathology and Laboratory Medicine, Cincinnati Children’s Hospital Medical Center, Cincinnati, OH, USA

^9^Department of Pediatrics, Cincinnati Children’s Hospital Medical Center, Cincinnati, OH, USA

^10^Department of Applied Biology and Chemical Technology, The Hong Kong Polytechnic University, Hung Hom, Kowloon, Hong Kong

^*^These two authors contributed equally to this work

Original article: Oncotarget. 2014; 5:1502–1514. 1502-1514. https://doi.org/10.18632/oncotarget.1609

